# Type, Timing, Frequency, and Durability of Outcome of Physical Therapy for Parkinson Disease: A Systematic Review and Meta-Analysis

**DOI:** 10.1001/jamanetworkopen.2023.24860

**Published:** 2023-07-21

**Authors:** Mario El Hayek, Janine Lemos Melo Lobo Jofili Lopes, Jennifer H. LeLaurin, Megan E. Gregory, Anna-Maria Abi Nehme, Patricia McCall-Junkin, Ka Loong Kelvin Au, Michael S. Okun, Ramzi G. Salloum

**Affiliations:** 1 Department of Health Outcomes and Biomedical Informatics, College of Medicine, University of Florida, Gainesville; 2 Norman Fixel Institute for Neurological Diseases, Department of Neurology, College of Medicine, University of Florida, Gainesville; 3 Health Science Center Library, University of Florida, Gainesville, Florida; 4 Department of Neurology, University of Kansas Medical Center, Kansas City

## Abstract

**Importance:**

Parkinson disease (PD) is a neurodegenerative syndrome affecting approximately 1% of the population older than 60 years, and a major goal of treatment is preservation of physical function through physical therapy (PT). Although PT outcomes for PD are well documented, aggregate information on the parameters of PT are needed to guide implementation.

**Objective:**

To evaluate current evidence on the types, timing, frequency, duration, and outcomes of PT regimens applied for PD.

**Data Sources:**

PubMed, Embase, Medline, and the Web of Science Core Collection were searched for articles published from January 1, 2000, to August 10, 2022. Search terms included terms related to Parkinson disease, PT interventions, and PT-related outcomes.

**Study Selection:**

Included studies were peer-reviewed randomized clinical trials available in English of any PT intervention for patients with PD that included PT-related outcomes. The Preferred Reporting Items for Systematic Reviews and Meta-analyses reporting guideline was followed.

**Data Extraction and Synthesis:**

Two reviewers extracted data and assessed quality using the Cochrane Risk of Bias Tool. Data were analyzed using a random-effects model.

**Main Outcomes and Measures:**

A meta-analysis compared outcomes of nonstandard PT vs standard PT and standard PT vs no intervention for Unified Parkinson’s Disease Rating Scale (UPDRS) score and measures of gait and balance.

**Results:**

A total of 46 trials with 3905 patients were included (range of mean ages, 61-77 years). Ten trials (22%) compared 2 types of nonstandard PT interventions; 26 (57%), nonstandard PT vs standard PT; and 10 (22%), PT vs no intervention. The most common nonconventional PT intervention was aquatic physiotherapy (5 trials [11%]). Durations of PT regimen ranged from 2 to 12 weeks in 39 trials (85%), and PT was most commonly performed with frequencies of either twice or 3 times weekly (27 [59%]). In most trials (39 [85%]), PT session length ranged from 30 to 60 minutes. Across trials, PT outcomes were reported for gait (14 trials [30%]), balance (10 [22%]), quality of life (3 [9%]), and cognition (1 [2%]). Approximately half of the trials (22 [48%]) documented durability of some level of benefit after completion of the prescribed regimen. Meta-analysis showed no significant difference for PT vs no intervention in UPDRS scores (standardized mean difference [SMD], −1.09; 95% CI, −2.50 to 0.33) or for nonstandard PT vs standard PT in measures of gait (SMD, 0.03; 95% CI, −0.53 to 0.59), balance (SMD, 0.54; 95% CI, −0.03 to 1.12), and UPDRS score (SMD, −0.49; 95% CI, −1.04 to 0.06). Meta-analytic regression of moderators revealed no significant differences in outcomes by frequency of PT per week (SMD, 0.17; 95% CI, –0.03 to 0.36).

**Conclusions and Relevance:**

The findings suggest that although a wide range of types and regimens of PT for PD have been tested, comparative effectiveness of different models of care and implementation strategies as well as long-term durability of their outcomes remain undetermined.

## Key Points

**Question:**

What is the optimal type, timing, frequency, and duration of physical therapy (PT) for Parkinson disease (PD)?

**Findings:**

This systematic review and meta-analysis of 46 randomized clinical trials of 3905 patients with PD found no differences between standard and nonstandard PT in gait, balance, and motor outcomes. Although many PT regimens were tested for PD, data were sparse on optimal type, timing, frequency, and duration and on the durability of outcomes.

**Meaning:**

Although PT for PD has been shown to be associated with mobility benefits and there are guidelines for implementation, research is needed to better define the ideal type, timing, frequency, and duration.

## Teaser

This systematic review and meta-analysis evaluates the type, timing, frequency, and duration of physical therapy for Parkinson disease and whether outcomes are durable over time.

## Introduction

Parkinson disease (PD) is a progressive neurodegenerative syndrome affecting approximately 1% of the population older than 60 years.^1^ Prevalence estimates for PD increased from 2.5 million in 1990 to approximately 6 million in 2016,^2^ and PD is now the fastest growing neurological disease worldwide.^3^ Additionally, a 2022 analysis revealed that PD prevalence is underestimated by approximately 50% and new cases are diagnosed every 6 minutes.^4^ The burden of a progressive PD diagnosis is usually accompanied by functional disability and impaired quality of life.^5^ Symptoms include mobility deficits, problems with transfers, progressive balance dysfunction, and significant challenges in walking.^6^ A multidisciplinary approach has been recommended by many experts,^7^ and physical therapy (PT) is considered a cornerstone of PD treatment plans. Despite published guidelines from the American Physical Therapy Association,^8^ implementation of long-term PT programs for PD has been hampered by availability of information.

One of the crucial treatment goals of PD is maintenance of mobility and physical function. This goal may possibly be achieved through the application of PT,^6^ a medical intervention that helps restore functional movement, such as standing, walking, or moving different body parts, that is delivered by physical therapists and/or movement experts. Multiple trials and systematic reviews have documented the benefits of PT for PD.^9,10^ However, previous reviews addressing PT for PD have focused on specific PT types and exercise modalities (eg, aquatic therapy^11^ or virtual reality^12^) rather than methods of administration and maintenance of outcomes. There is a paucity of comprehensive studies examining PT modalities and methods for application and maintenance of PT. Agreement on how to administer PT for PD has been elusive, and potentially important variables, such as PT timing, frequency, and duration, may prove to be important to maintenance of outcomes.^13^ To address these knowledge gaps, we sought to evaluate the current evidence on methods of PT delivery for PD inclusive of type, timing, and frequency as well as the durability of outcome.

## Methods

### Identification of Eligible Publications

To be included in this systematic review and meta-analysis, studies must have been peer-reviewed and available in English. Eligible studies were randomized clinical trials published between January 1, 2000, and August 10, 2022, that included people with PD and compared a PT intervention with another intervention or no intervention. Duplicate abstracts were excluded, as were abstracts describing reviews, editorials, commentaries, protocols, conference abstracts, and dissertations. Articles from non–peer-reviewed journals and articles with untranslated full texts written in a language other than English were excluded. The review protocol was registered in PROSPERO. The study followed the Preferred Reporting Items for Systematic Reviews and Meta-analyses (PRISMA) reporting guideline.

### Search Strategy

An electronic search of the literature was conducted in multiple databases: PubMed, Embase, Medline, and the Web of Science Core Collection. Search strategies included terms about the study population (*Parkinson disease*, *idiopathic Parkinson disease*, and *Parkinson*), the intervention (eg, *physical therapy*, *physical therapy modalities*, *resistance training*, *strength training*, and *weight training*), and some PT-related outcomes (ie, *quality of life*, *activities of daily living*, and *motor skills*). The full search strategy is available in eAppendix 1 in Supplement 1. We combined text and, where appropriate, Medical Subject Headings terms from all 3 categories. All sources were last searched on August 10, 2022.

### Screening and Data Extraction

Study screening and data extraction were performed in Covidence. All abstracts and full-text articles were screened by 3 independent reviewers (M.E.H., J.L.M.L.J.L., and A.-M.A.N.) according to the inclusion and exclusion criteria. Conflicts were resolved by discussion and consensus among the 3 reviewers. Data were extracted from each article by 1 of the 3 reviewers, with another of the reviewers independently auditing the article to ensure accuracy of data extraction. The following variables were extracted: title, publication year, country, hospital, setting, funding sources, start and end date, primary and secondary aims, total number of participants, baseline population characteristics (age, gender, race and ethnicity, and educational level), PD characteristics (disease duration, baseline disease severity, medications, and cognitive impairment), PT characteristics (type, frequency, time intervals, session length, and duration), and PT outcomes (motor performance, mobility and gait, balance, and recent falls). Quality assessment was performed by 4 independent reviewers (M.E.H., J.L.M.L.J.L., J.H.L., and A.-M.A.N.) using the Cochrane Risk of Bias Tool, a standardized tool used to evaluate research bias in systematic reviews.^14^

### Statistical Analysis

After exclusion of all trials with high risk of bias, a meta-analysis was separately conducted for studies comparing nonstandard vs standard PT and studies comparing standard PT vs no intervention. Trials comparing different types of nonstandard PT were excluded due to the heterogeneity of the various types of nonstandard PT. Trials with a design of 3 or more arms were excluded. Included studies must have stated either the effect sizes or the means and SDs of the outcome in both the experimental and the control groups. The meta-analysis was conducted using Review Manager, version 5.4.^15^ We implemented the inverse variance statistical method, with an analysis model of random effects and an effect measure of standardized mean difference (SMD), and 95% CIs were considered. The most commonly reported outcomes were categorized as follows: the Unified Parkinson’s Disease Rating Scale (UPDRS), measures of gait (timed up and go test and 10-m walking test), and measures of balance (Berg Balance Scale [BBS] and Mini Balance Evaluation System Test [mini-BESTest]). Other outcome types were reported too infrequently to be included in the meta-analysis. Across all studies in the meta-analysis, we conducted follow-up meta-regressions to assess whether duration of PT, frequency per week, and total number of PT sessions (computed by multiplying the 2 prior values) were significant moderators. We also dichotomized duration into subgroups of less than 12 or 12 or more weeks and of 6 or fewer or more than 6 weeks. For the meta-regressions, the metafor package in R, version 4.2 (R Project for Statistical Computing) was used.

## Results

### Study Selection

**Figure 1.**
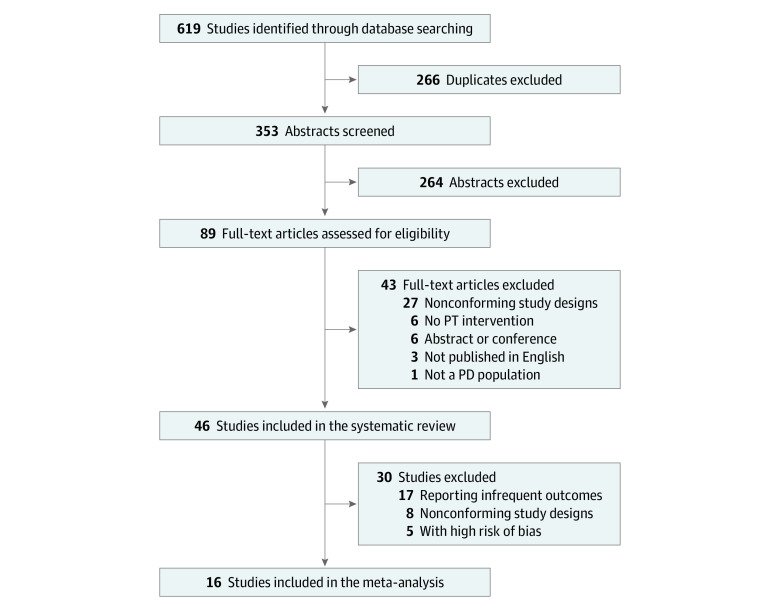
PRISMA Flow Diagram PD indicates Parkinson disease; PT, physical therapy.

The search returned 353 abstracts after excluding all duplicates; 264 abstracts were excluded based on the eligibility criteria. Of the remaining 89 studies, 43 were excluded for the following reasons: nonconforming study design (n = 27), no PT intervention (n = 6), abstract only or conference presentation (n = 6), article not published in English (n = 3), and/or no PD patient population (n = 1) (Figure 1). The remaining 46 studies with a total of 3905 patients were included in the review.

### Included Studies

Most studies (27 [59%])^16,17,18,19,20,21,22,23,24,25,26,27,28,29,30,31,32,33,34,35,36,37,38,39,40,41,42^ were published after 2016 (Table 1 and eAppendix 2 in Supplement 1). More than half of the trials were conducted in Italy (19 [41%]),^17,19,20,21,23,24,26,29,30,31,34,40,41,46,49,51,52,59,60^ Brazil (5 [11%]),^18,22,27,44,45^ or the US (5 [11%]).^39,47,48,54,56^ A fraction of the remaining trials was conducted in the UK (4 [9%]).^16,35,53,61^ The number of participants per trial ranged between 15^47^ and 762^16^ participants. Thirty-two studies (70%) involved 50 participants or less,^17,18,19,20,21,22,23,24,25,26,27,28,29,32,33,37,39,40,41,42,44,45,46,47,49,51,54,55,58,59,60,61^ and 8 studies (17%) enrolled more than 100 participants.^16,35,36,38,50,53,57,62^ Only 2 studies had sample sizes exceeding 500.^16,57^ The mean age of participants ranged from 61^17^ to 77^18^ years. With respect to baseline disease severity, more than half of the trials (25 [54%])^17,21,24,25,27,29,30,31,38,39,40,41,42,43,44,45,46,48,50,51,53,56,58,59,60^ included participants with a Hoehn and Yahr stage between 2 and 3. Overall, 44 trials (96%)^16,17,18,20,21,22,23,24,25,26,27,28,29,30,31,32,33,34,35,36,37,38,39,40,41,42,43,44,45,46,47,48,49,50,51,52,53,54,55,56,57,58,59,60^ used a parallel design and 2 (5%) used a crossover design.^19,61^ Of the 44 parallel design trials, 43 (98%)^16,17,18,21,22,23,24,25,26,27,28,29,30,31,32,33,34,35,36,37,38,39,40,41,42,43,44,45,46,47,48,49,50,51,52,53,54,55,56,57,58,59,60^ were single-blinded and only 1 (2%) was double-blinded.^20^ Overall, 45 trials (98%)^16,17,18,19,20,22,23,24,25,26,27,28,29,30,31,32,33,34,35,36,37,38,39,40,41,42,43,44,45,46,47,48,49,50,51,52,53,54,55,56,57,58,59,60,61^ were conducted in an outpatient setting and only 1 (2%) in an inpatient setting.^21^

**Table 1.** Summary of the 46 Included Trials

Source (country)	Sample size (mean age, y) [H&Y stage]	Intervention	Control	Primary outcome	Main findings
Acarer et al,^43^ 2015 (Turkey)	60 (67) [2-3]	Vestibular rehabilitation	No intervention	Not specified	Vestibular rehabilitation was effective for improving balance (BBS, ABC, DGI) after 8 wk
Agosti et al,^23^ 2016 (Italy)	20 (62.9) [NA]^a^	GPR	No intervention	Kinematic parameters of gait, UPDRS III	GPR group had significant improvement in kinematic parameters of gait and UPDRS III persisting after 12 wk
Akhila et al,^33^ 2022 (India)	32 (70.8) [1.5-3.0]^a^	Task-related trunk training	Standard PT	Mini-BESTest	Task-related trunk training appeared more beneficial than standard physiotherapy in balance capacity after 6 wk
Alagumoorthi et al,^36^ 2022 (India)	192 (69.7) [1.5-3.0]^a^	Wii training	Balance training	Number of participants who fell	Wii group significantly improved over control group at 12th and 36th week in number of fallers, fall rate, and bodily discomfort component of PDQ-39
Au et al,^39^ 2022 (US)	22 (69.7) [2.1]^a^	Spaced PT	Burst PT	TUG	Spaced PT group had stability of the TUG at 6 mo; burst group had significant worsening once PT was discontinued after 6 wk
Bonnì et al,^20^ 2019 (Italy)	16 (71.8) [NA]^a^	Blindfolded balance training	Standard PT	Gait, neurophysiological	Decrease of stance and double stance phase and increase of swing phase with respect to gait cycle in BBT group vs PT group after 2 wk
Capato et al,^18^ 2020 (Brazil)	35 (77) [4]^a^	PT, RAS	Standard PT	Mini-BESTest	RAS and regular balance training improved balance in patients with PD after 5 wk, but long-term effects, up to 6 mo, were sustained only in the RAS group
Capecci et al,^44^ 2014 (Brazil)	20 (73.4) [3]	Postural rehabilitation or postural rehabilitation plus kinesiology taping	No intervention	BBS, TUG, trunk bending	All treated patients showed significant improvement in trunk posture, gait, and balance at 1 mo; benefits persisted at 2 mo for all measures except lateral trunk bend
Carpinella et al,^29^ 2017 (Italy)	42 (73) [2.7]^a^	Balance and gait training with gamepad	Structured PT	BBS, 10MWT	Gamepad-based training was superior to PT in improving BBS performance and retaining it for 1 mo; 10MWT data were comparable between groups after training
Carvalho et al,^45^ 2015 (Brazil)	22 (64.1) [2.1]	Strength training or aerobic training	Regular PT	UPDRS III	Strength and aerobic training groups had UPDRS III improved by 27.5% and 35%, respectively; regular PT improved by 2.9% after 12 wk
Cholewa et al,^28^ 2017 (Poland)	42 (61.3) [3.2]^a^	Rehabilitation exercises	No intervention	Gait speed, mean step length, step frequency	Rehabilitation group showed significant improvement in gait speed (12.35%), mean step length (18.00%), and frequency step (2.40%) vs control group after 9 mo
Clarke et al,^16^ 2016 (UK)	762 (70) [NA]^a^	PT	No intervention	NEADL scale score	PT and OT were not associated with clinically meaningful improvements in ADLs or QOL at 3 mo
Clerici et al,^30^ 2019 (Italy)	60 (67) [2.7]	AT plus motor cognitive rehabilitation	Motor cognitive rehabilitation	FOG	MIRT improved FOG at 4 wk; AT added no further benefits
de Natale et al,^40^ 2017 (Italy)	16 (67) [2.5]^a^	DT	Traditional PT	9-HPT	DT had motor and cognitive outcomes significantly improved at 2 wk of treatment and retained after follow-up of 8 wk vs TR
Dipasquale et al,^24^ 2017 (Italy)	40 (69.9) [2]^a^	Standard PT	General exercises	FIM, HRS, TUG, UPDRS	PT seemed more effective than a generic exercise program at 4 mo in patients with H&Y stage II PD
Feng et al,^25^ 2019 (China)	28 (67.5) [3.1]^a^	Virtual reality training	Conventional PT	BBS, TUG, UPDRS III, FGA	12 wk of VR training improved gait and balance vs conventional PT (significant for BBS, TUG, and FGA but not UPDRS III)
Ferrazzoli et al,^26^ 2018 (Italy)	36 (64.5) [NA]	Multidisciplinary intensive rehabilitation plus rotiogotine	Rotigotine only	UPDRS III	During 18 mo, no differences in UPDRS III between the 2 groups, but MIRT plus rotigotine group improved in 6MWT and TUG
Ferreira et al,^22^ 2018 (Brazil)	35 (64.1) [1]^a^	Resistance training	No intervention	Anxiety symptoms and QOL	Significant reduction in anxiety symptoms and increase in QOL after 24 wk
Frazzitta et al,^46^ 2012 (Italy)	50 (72) [3]^a^	IRT	Walking instructions at home	UPDRS, UPDRS II, UPDRS III	IRT group preserved UPDRS, UPDRS II, and UPDRS III values over 1 y; control group had a significant increase in those variables in same period
Frisaldi et al,^17^ 2021 (Italy)	38 (60.7) [2]^a^	Conventional PT and dance therapy	Conventional PT	MDS-UPDRS III	DT method was more effective than intensive program of conventional PT in improving motor impairment in patients with mild PD after 5 wk
de Oliveira Gondim et al,^27^ 2017 (Brazil)	28 (65) [2]^a^	Individualized orientation and phone monitoring	Usual exercise instructions	ADLs, UPDRS-motor, PDQ-39	Significant improvement in ADL and UPDRS-motor, PDQ-39 total and dimensions mobility, emotional well-being, and bodily discomfort in the experimental vs control group at 12 wk
Hirsch et al,^47^ 2003 (US)	15 (70.8) [1.8]^a^	Balance and resistance training	Balance training	SOT, muscle strength	Both types of training improved SOT performance for at least 4 wk; effect was larger in combined group
King et al,^48^ 2015 (US)	58 (63.9) [2.4]	Home exercise program, individual PT, or group class	No intervention	7-PPT	Only the individual group significantly improved in the 7-PPT after 4 wk, mostly in functional and balance measures
Marchese et al,^49^ 2000 (Italy)	20 (65) [1.5-3]^a^	Cued PT	Noncued PT	Not specified	Both groups had significant reduction of ADLs and motor sections of UPDRS after 6 wk of rehabilitation; this improvement largely faded in the noncued group but was still significant in the cued group after 12 wk
McGinley et al,^50^ 2012 (Australia)	210 (67.9) [2.5]	Progressive strength training or movement strategy training (both with fall prevention)	Life skills	Feasibility of rehabilitation and PT programs	All 3 programs proved feasible, suggesting they may be safely implemented for people with PD in community-based clinical practice
Modugno et al,^51^ 2010 (Italy)	24 (62) [3]^a^	Active theater program	Standard PT	Clinical disability and QOL	Theater group showed progressive significant improvements at the end of the third year in all clinical scales
Monticone et al,^52^ 2015 (Italy)	70 (74.1) [2.5-4]^a^	Multidisciplinary rehabilitation care	General PT	MDS-UPDRS III	Multidisciplinary rehabilitation care positively changed the course of motor impairment, balance, and activities of daily living; effects lasted for at least 1 y after the intervention
Morris et al,^38^ 2017 (Australia)	133 (71) [2]	Progressive resistance strength training	Nonspecific life skills training	Rate of falls	Home program of strength and movement strategy training and falls education did not prevent falls in 12 mo when applied at the study dose
Nieuwboer et al,^53^ 2007 (UK, Belgium, the Netherlands)	153 (67.5) [2.5]^a^	Home cueing program followed by 3 wk with no training	3 wk of no training followed by home cueing program	Posture and gait score	Significant improvements after intervention on the posture and gait scores; severity of freezing was reduced in freezers only; gait speed, step length, and timed balance tests improved in the full cohort; effects of intervention were reduced at 6-wk follow-up
Pazzaglia et al,^34^ 2020 (Italy)	51 (72) [NA]^a^	VR rehabilitation program	Conventional rehabilitation program	BBS	VR rehabilitation program led to increase in BBS and DGI scores vs conventional rehabilitation program at 6 wk
Pelosin et al,^31^ 2018 (Italy)	70 (70.4) [2.4]^a^	Action observation therapy	Standard PT	FOG	AOT was feasible, safe, and efficacious in improving FOG at 5 wk
Pérez-de la Cruz,^37^ 2018 (Spain)	29 (65.9) [NA]^a^	Aquatic Ai Chi program	Dry land conventional PT	TUG, PDQ-39-SS, LS, VAS, FTSTS, Yesavage test	Ai Chi program reduced limb bradykinesia and joint rigidity, decreased pain, and improved self-reported QOL at 11 wk in patients with H&Y stage 1-3 PD
Qutubuddin et al,^54^ 2007 (US)	22 (71.2) [NA]^a^	CDP therapy	Balance PT	BBS, CDP variables	No differences found between treatment groups, but both groups demonstrated improvement on selected outcome measures
Raciti et al,^21^ 2022 (Italy)	30 (65.7) [2]	Experimental robotic therapy	Conventional PT	9-HPT	Exoskeleton-assisted therapy may be a safe and effective strategy for delivering highly intensive and repetitive training
San Martín Valenzuela et al,^32^ 2020 (Spain)	40 (66.4) [1-3]^a^	Dual task training	Single task training	Velocity and spatiotemporal parameters of gait	Dual-task group demonstrated improved velocity and stride length time in all assessment conditions after 5 wk of training and improved perceived QOL
Chivers Seymour,^35^ 2019 (UK, Belgium)	474 (71) [1-4]^a^	PDSAFE	DVD plus counseling	Risk of repeat falling	PDSAFE did not reduce falling at 6 mo
Tamir et al,^55^ 2007 (Israel)	23 (67.4) [NA]^a^	Combination of imagery and physical practice	Physical practice	Not specified	Significantly faster performance of movement sequences at 12 wk and higher gains in mental and motor subsets of the UPDRS and in cognitive tests vs the control group
Tickle-Degnen et al,^56^ 2010 (US)	117 (66.3) [2-3]	18-h Rehabilitation or 27-h rehabilitation	No intervention	HRQOL	Both groups had significant beneficial effects in HRQOL at 6 wk, with benefits persisting at 2- and 6-mo follow-up
van Nimwegen et al,^57^ 2013 (the Netherlands)	586 (65.1) [1-3]^a^	ParkFit program	Standard PT	Activity diary and monitor	ParkFit did not change the overall volume of physical activities in older, sedentary patients with PD
Varalta et al,^19^ 2021 (Italy)	20 (70.8) [NA]^a^	Consecutive PT and cognitive training	Standard PT	MOCA, UPDRS III	Improvements in walking abilities were noted after both interventions, but only the patients treated with consecutive training showed better performance on functional mobility and memory tasks
Vivas et al,^58^ 2011 (Spain)	12 (65.7) [2.67]^a^	Water-based therapy	Land-based therapy	Not specified	Main effect of both therapies seen at 4 wk for the FRT; only the AT group improved in the BBS and the UPDRS
Volpe et al,^59^ 2014 (Italy)	34 (68) [2.82]^a^	Hydrotherapy	Land-based standard rehabilitation	Center of the pressure sway area	Better improvement in patients who underwent hydrotherapy than land-based therapy in the center of pressure sway area closed eyes, BBS, ABC, Falls Efficacy Scale, PDQ-39, and falls diary at 2 mo
Volpe et al,^41^ 2017 (Italy)	30 (70.6) [2-3]^a^	Water-based physiotherapy	Non–water-based physiotherapy	Cervical and dorsal flexion, lateral trunk inclination	After 8 wk of treatment, only the water-based group showed a significant improvement of trunk posture with a significant reduction of cervical flexion, dorsal flexion, and lateral inclination of the trunk
Volpe et al,^60^ 2013 (Italy)	24 (61.6) [2-3]^a^	Irish set dancing classes plus home program	Standard PT	UPDRS	Irish dancing and PT were safe and feasible, with good adherence over 6 mo; although improvements were made in both groups, the dance group showed results superior to standard physiotherapy for FOG, balance, and motor disability
Wade et al,^61^ 2003 (UK)	24 (71.3) [NA]	Active rehabilitation	No intervention	Not specified	Patients with PD declined significantly over 6 mo, but short-term multidisciplinary rehabilitation may improve mobility
Wróblewska et al,^42^ 2019 (Poland)	40 (72.1) [2-3]^a^	Nordic walking	No intervention	FOG	NW training had a beneficial effect on FOG in PD at 3 mo that persisted to 6 mo

Abbreviations: 7-PPT, 7-item physical performance test; 9-HPT, hole peg test; 10MWT, 10-m walking test; ABC, activities-specific balance confidence scale; ADLs, activities of daily living; AOT, action observation therapy; AT, aquatic therapy; BBS, Berg Balance Scale; CDP, computerized dynamic posturography; DGI, dynamic gait index; DT, dance therapy; FGA, functional gait assessment; FIM, functional independence measure; FOG, freezing of gait; FRT, functional reach test; FTSTS, 5 times sit-to-stand test; GPR, global postural reeducation; H&Y, Hoehn and Yahr; HRQOL, health-related quality of life; HRS, Hamilton Rating Scale; IRT, intensive rehabilitation treatment; LS, leg standing; MDS-UPDRS III, Movement Disorder Society Sponsored Revision of the Unified Parkinson Disease Rating Scale; mini-BESTest, Mini Balance Evaluation System Test; MIRT, multidisciplinary intensive rehabilitation treatment; MOCA, Montreal Cognitive Assessment Test; NA, not applicable; NEADL, Nottingham Extended Activities of Daily Living; NW, Nordic walking; OT, occupational therapy; PD, Parkinson disease; PDQ-39, Parkinson Disease Questionnaire; PDQ-39-SS, Parkinson Disease Questionnaire–Social Support; PT, physical therapy; QOL, quality of life; RAS, rhythmical auditory stimulation; SOT, sensory orientation test; TR, traditional rehabilitation; TUG, timed up and go test; UPDRS, Unified Parkinson’s Disease Rating Scale; UPDRS II, Unified Parkinson Disease Rating Scale for activities of daily living; UPDRS III, Unified Parkinson Disease Rating Scale for motor skills; VAS, visual analog scale; VR, virtual reality.

^a^ Variables of the experimental group were included in the table if the mean age and/or baseline H&Y stage of the trial participants was not mentioned.

### Outcome Measures

The primary outcomes varied across the included trials. Some trials measured patient-reported outcomes (eg, quality of life),^16,22,51^ and others targeted functional outcomes (eg, 10-m walking test).^29^ The most common primary outcome was the UPDRS, including its different components (ie, UPDRS II [activities of daily living], UPDRS III [motor skills], and Movement Disorder Society Sponsored Revision of the UPDRS). The UPDRS measures were used in 14 studies (30%).^17,19,23,24,25,26,27,43,45,46,52,55,58,60^ Other common primary outcomes included gait (14 studies [30%]),^20,23,24,25,28,29,30,31,32,42,44,53,54,58^ using measurements such as the 10-m walking test,^58^ the timed up and go test,^44^ or other gait parameters.^20^ One other category, in 10 studies (22%), addressed balance measures such as the BBS or the mini-BESTest.^18,25,29,33,34,43,44,54,55,58^ Other outcomes included falls or postural stability (4 studies [9%]),^35,36,43,53^ the Parkinson Disease Questionnaire-39 (4 [9%]),^27,37,43,56^ other quality of life measures (3 [7%]),^22,43,51^ and cognition (1 [2%]).^40^

The included trials used 1 of 3 types of comparison groups. Some trials (10 [22%]^29,30,32,35,36,39,47,53,54,55^) compared 2 different types of PT (eg, dance therapy vs conventional PT^17^), while 10 (22%)^16,22,23,28,42,43,44,48,56,61^ compared PT with a no-intervention control group.^28,49^ The remaining studies (26 [57%]^17,18,19,20,21,24,25,26,27,31,33,34,37,38,40,41,45,46,49,50,51,52,57,58,59,60^) compared PT with general exercise.^24,46^ One trial included PT delivered in a group setting.^48^

When comparing 2 different types of PT, it was not uncommon to measure UPDRS and/or balance as primary outcomes. In fact, multiple studies showed that 1 type of PT was superior to another in improving balance in patients with PD.^18,25,29,33,34^ For example, virtual reality training had significantly better balance outcomes compared with standard PT,^34^ and task-related trunk training with emphasis on spine exercises proved to be superior to standard PT based on the mini-BESTest.^33^ One trial showed that water-based PT improved balance (BBS) compared with land-based PT in patients with PD.^58^ Safety and feasibility of PT interventions in patients with PD were assessed in 2 different trials, and both had affirmative results.^21,50^ However, 5 of the 46 trials (11%) did not find a difference in their primary outcome(s).^16,26,35,38,57^ Two of those trials targeted falls in their outcomes.^35,38^

### Physical Therapy Regimens

Physical therapy modalities varied considerably across studies (Table 2). Twenty-one trials (46%) described 1 of their comparison arms as standard or conventional PT. Generally, standard or conventional PT is delivered by a physical therapist in a regular outpatient center and consists of general body movements and exercises. Any variation in the setting, type, intensity, or mode of delivery was categorized as nonstandard or nonconventional. Those interventions included water-based PT (5 studies [11%]),^30,37,41,59,60^ followed by multidisciplinary therapy (4 [9%])^18,19,26,52^; balance, resistance, or strength training (4 [9%])^22,38,45,47^; and dance therapy (3 [7%]).^17,40,60^ Other interventions included virtual reality rehabilitation (2 [4%]),^25,34^ walking (2 [4%]),^35,42^ cueing PT (2 [4%]),^49,53^ and game-based rehabilitation (2 [4%]).^29,36^

**Table 2.** Summary of the Type, Duration, Frequency, Session Length, and Durability of Outcome of the Physical Therapy Regimen in the Intervention and Control Groups

Source	Intervention group			Control group			Durability of outcome
	Frequency	Session length	Regimen duration	Frequency	Session length	Regimen duration	
Acarer et al,^43^ 2015	NA	30-40 min	8 wk	NA	NA	NA	NA
Agosti et al,^23^ 2016	3 Times/wk	40 min	4 wk	NA	NA	NA	4 and 8 wk
Akhila et al,^33^ 2022	3 Times/wk	60 min	6 wk	3 Times/wk	60 min	6 wk	NA
Alagumoorthi et al,^36^ 2022	3 Times/wk	30-40 min	12 wk	3 Times/wk	30-40 min	12 wk	24 wk
Au et al,^39^ 2022	2 Times/wk	NA	6 wk	1 Time every 2 wk	NA	6 mo	18 wk
Bonnì et al,^20^ 2019	5 Times/wk	40 min	2 wk	5 Times/wk	45 min	2 wk	NA
Capato et al,^18^ 2020	2 Times/wk	45 min	5 wk	2 Times/wk	45 min	5 wk	1 and 6 mo
Capecci et al,^44^ 2014	3 Times/wk	40 min	4 wk	NA	NA	NA	4 wk
Carpinella et al,^29^ 2017	3 Times/wk	45 min	7 wk	3 Times/wk	45 min	7 wk	4 wk
Carvalho et al,^45^ 2015	2 Times/wk	40 min	12 wk	NA	NA	NA	NA
Cholewa et al,^28^ 2017	2 Times/wk	60 min	36 mo	NA	NA	NA	NA
Clarke et al,^16^ 2016	Median sessions, 4	58 min	8 wk	NA	NA	NA	NA
Clerici et al,^30^ 2019	4 Times/d for 5 d, PT the 6th day	60 min	4 wk	4 Times/d for 5 d, PT on the 6th day, 3 AT weekly	60 min	4 wk	NA
De Natale et al,^40^ 2017	2 Times/wk	60 min	10 wk	2 Times/wk	60 min	10 wk	8 wk
Dipasquale et al,^24^ 2017	2 Times/wk	60 min	4 mo	2 Times/wk	60 min	4 mo	135 d
Feng et al,^25^ 2019	5 d/wk	45 min	12 wk	5 Times/wk	45 min	12 wk	NA
Ferrazzoli et al,^26^ 2018	4 Sessions for 5 d plus 1 h of exercise on the 6th day	60 min	4 wk	NA	NA	NA	6, 12, and 18 mo
Ferreira et al,^22^ 2018	2 Times/wk	30-40 min	24 wk	NA	NA	NA	NA
Frazzitta et al,^46^ 2012	3 Times daily, 5 d/wk	60 min	4 wk	NA	NA	NA	NA
Frisaldi et al,^17^ 2021	3 Times/wk	60 min	5 wk	3 Times/wk	60 min	5 wk	NA
de Oliveira Gondim et al,^27^ 2017	3 Times/wk	Up to 1 h	12 wk	3 Times/wk	Up to 1 h	12 wk	NA
Hirsch et al,^47^ 2003	3 Times/wk	15 min Resistance, 30 min balance	10 wk	3 Times/wk	30 min	10 wk	4 wk
King et al,^48^ 2015	3 Times/wk	60 min	6 wk	3 Times/wk	60 min	6 wk	NA
Marchese et al,^49^ 2000	3 Times/wk	60 min	6 wk	3 Times/wk	60 min	6 wk	6 wk
McGinley et al,^50^ 2012	1 Time/wk	120 min	8 wk	1 Times/wk	120 min	8 wk	NA
Modugno et al,^51^ 2010	2 Consecutive days either 1 or 2 times per mo	6 h	3 y	3 Times/wk	2 to 3 h	3 y	NA
Monticone et al,^52^ 2015	Daily	90 min	8 wk	Daily	90 min	8 wk	12 mo
Morris et al,^38^ 2017	2 Times/wk	60 min	6 wk	2 Times/week	60 min	6 wk	12 mo
Nieuwboer et al,^53^ 2007	9 Sessions over 3 wk	30 min	3 wk	9 Sessions over 3 wk	30 min	3 wk	6 wk
Pazzaglia et al,^34^ 2020	3 Times/wk	40 min	6 wk	3 Times/wk	40 min	6 wk	NA
Pelosin et al,^31^ 2018	2 Times/wk	45 min	5 wk	2 Times/wk	45 min	5 wk	4 wk
Pérez-de la Cruz,^37^ 2018	2 Times/wk	45 min	11 wk	2 Times/wk	45 min	11 wk	4 wk
Qutubuddin et al,^54^ 2007	2 Times/wk	30 min	4 wk	2 Times/wk	30 min	4 wk	NA
Raciti et al,^21^ 2022	6 Times/wk	45 min for each arm (90 min total)	8 wk	6 Times/wk	45 min	8 wk	NA
San Martín Valenzuela et al,^32^ 2020	2 Times/wk	60 min	10 wk	2 Times/wk	60 min	10 wk	8 wk
Chivers Seymour et al,^35^ 2019	12 Sessions in 6 mo plus daily exercise	1-1.5 h Supervised session, 30 min exercise	72 wk	1 Session on fall avoidance	NA	NA	6 mo
Tamir et al,^55^ 2007	2 Times/wk	1 h	12 wk	2 Times/wk	1 h	12 wk	NA
Tickle-Degnen et al,^56^ 2010	3 Times/wk	90 min	6 wk	2 Times/wk	90 min	6 wk	2 and 18 wk
van Nimwegen et al,^57^ 2013	Maximum of 35 sessions/y	NA	12 mo	Maximum 35 sessions/y	NA	12 mo	NA
Varalta et al,^19^ 2021	PT once weekly; cognitive once weekly	50 min	12 wk	2 Times/wk	50 min	12 wk	NA
Vivas et al,^58^ 2011	2 Times/wk	45 min	4 wk	2 Times/wk	45 min	4 wk	17 d
Volpe et al,^59^ 2014	5 Times/wk	60 min	8 wk	5 Times/wk	60 min	8 wk	NA
Volpe et al,^41^ 2017	5 Times/wk	60 min	8 wk	5 Times/wk	60 min	8 wk	8 wk
Volpe et al,^60^ 2013	1 Time/wk	90 min	24 wk	1 Time/wk	90 min	24 wk	NA
Wade et al,^61^ 2003	1 Time/wk	NA	6 wk	1 Time/wk	NA	6 wk	NA
Wróblewska et al,^42^ 2019	2 Times/wk	60 min	12 wk	NA	NA	NA	3 mo

Abbreviations: AT, aquatic therapy; NA, not available.

Physical therapy regimens differed in duration, frequency, and session length. The total duration ranged from 2 weeks^20^ to 3 years,^28,51^ whereas most studies had regimen durations of 2 to 12 weeks (39 [85%]^16,17,18,19,20,21,23,25,26,27,29,30,31,32,33,34,36,37,38,39,40,41,42,43,44,45,46,47,48,49,50,52,53,54,55,56,58,59,61^). Frequency ranged from once weekly^60,61^ to daily^52^; however, most of the trials had frequencies of either 2 or 3 times weekly (27 studies [59%]).^17,18,22,23,24,27,28,29,31,32,33,34,36,37,38,39,40,42,44,45,47,48,49,54,55,56,58^ Only 9 studies (20%) had frequencies greater than or equal to 5 times weekly.^20,21,25,30,35,41,46,52,59^ The session lengths were somewhat similar, with 40 trials (87%) reporting sessions that lasted from 30 to 60 minutes.^16,17,18,19,20,22,23,24,25,26,27,28,29,30,31,32,33,34,35,36,37,38,39,40,41,42,43,44,45,46,47,48,49,53,54,57,58,59,60,61^ Of the other 6 trials (13%), 4 had session lengths of 90 minutes,^35,52,56,60^ 1 had a session length of 120 minutes,^50^ and 1 had sessions lasting for 6 hours.^51^ Only 1 trial had 1 arm that administered PT in group sessions^48^; all other trials administered PT individually.

### Durability of Outcome

We investigated durability of the outcome of the PT interventions (ie, whether the observed outcome was maintained after PT was discontinued). We identified all trials that followed up patients for a minimum of 1 visit after the end of the intervention period. About half of the included trials (22 [48%]) addressed this issue.^18,23,24,26,29,31,32,35,36,37,38,39,40,41,42,44,47,49,52,53,56,58^ Patients receiving multiple cycles of PT with follow-up after each cycle were not included in the subgroup evaluation. The follow-up intervals, measured from the end of the PT intervention, are included in Table 2. Follow-up periods ranged from 17 days^58^ to 18 months.^26^ Most of the 22 trials had 1 maintenance follow-up visit (18 [82%]).^24,29,31,32,35,36,37,38,39,40,41,42,44,47,49,52,53,58^ Some trials had 2 maintenance follow-up visits (3 [14%]),^18,23,56^ and only 1 trial (5%) had 3 visits.^26^ The findings at each follow-up interval are included in Table 1. Seventeen of the 22 trials (77%) reported that the observed improvement at the end of treatment was durable until the subsequent follow-up.^18,23,24,29,31,32,36,37,40,41,42,44,47,49,52,56,58^ The remaining 5 trials (23%) revealed worsening of outcomes after stopping PT^39,53^ or that the PT intervention group lost durability of outcome over time.^26,35,38^

### Quality Assessment

Quality assessment was conducted using the Cochrane Risk of Bias Tool.^14^ Every section of the assessment tool was divided into high risk of bias, low risk of bias, or some concerns. Overall, 17 articles (37%) had a low risk of bias,^16,19,24,26,30,31,32,35,37,38,39,41,48,49,59,60,61^ 24 (52%) had some concerns,^17,18,20,21,22,23,25,27,29,33,34,40,44,45,46,50,51,52,53,54,55,56,57,58^ and 5 (11%) had a high risk of bias^28,36,42,43,47^(eAppendix 3 in Supplement 1). Studies were rated with high risk due to bias in the measurement of the outcome,^28,47^ deviations from intended interventions,^28,42^ or selection of the reported result.^30,43^ Studies classified as having some concerns mainly had insufficient reporting on the randomization process or on deviations from the intended interventions.

### Meta-Analysis

**Figure 2.**
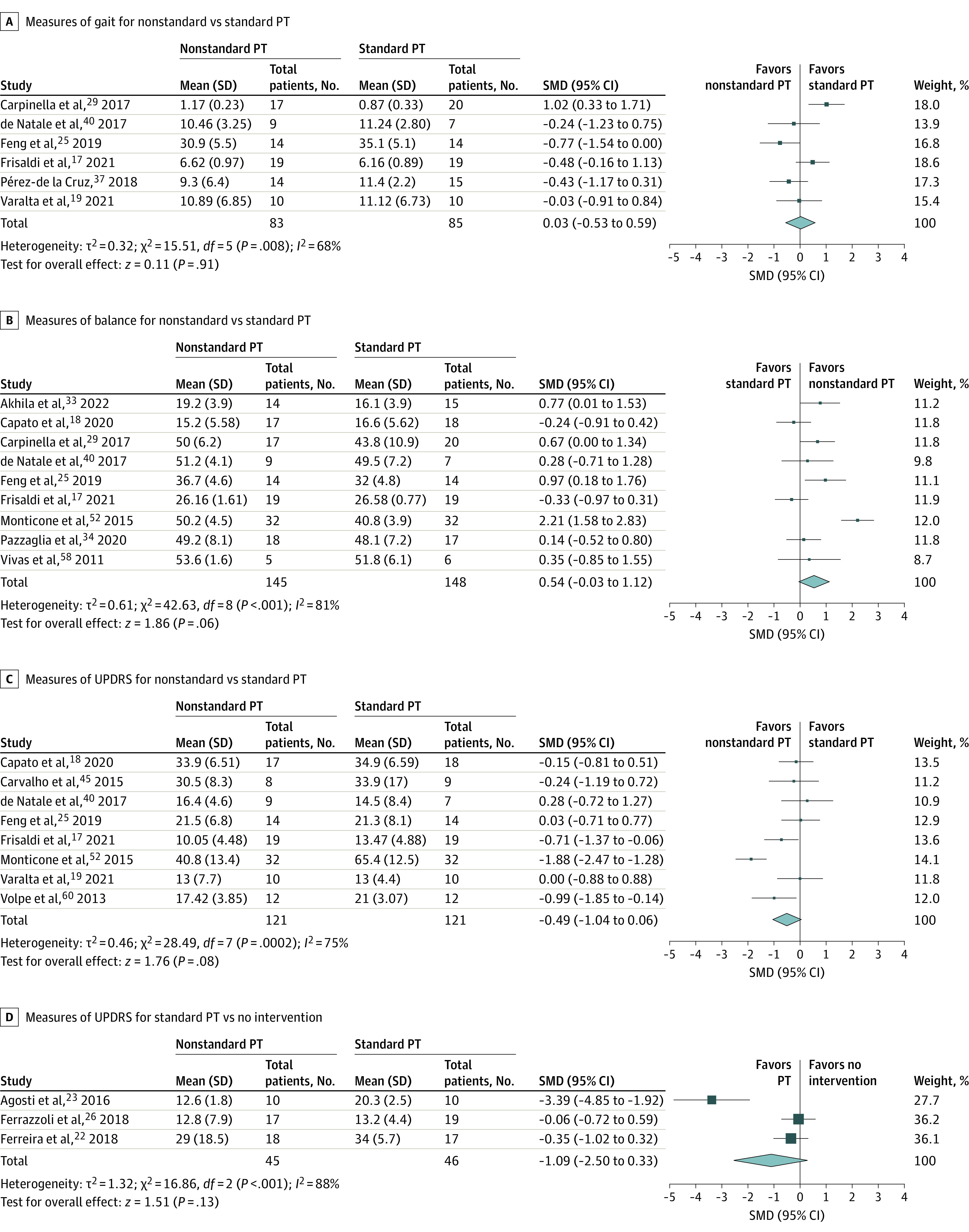
Forest Plots of Measures of Gait, Balance, and Unified Parkinson Disease Rating Scale (UPDRS) Scores Gait was assessed by the 10-m walk test and timed up and go test and balance by the Berg Balance Scale and Mini Balance Evaluation System Test. A random-effects model was used with inverse variance. Squares represent standardized mean differences (SMDs), with horizontal lines representing 95% CIs. Diamonds represent pooled SMDs, with outer points of the diamonds representing 95% CIs. PT indicates physical therapy.

There were enough studies to meta-analyze all 3 categories of outcomes when comparing nonstandard vs standard PT (*k* = 6 for gait,^17,19,25,29,37,40^ *k* = 9 for balance,^17,18,25,29,33,34,40,52,58^ and *k* = 8 for UPDRS^17,18,19,25,40,45,52,60^). However, when comparing standard PT vs no intervention, only UPDRS measures could be analyzed (*k* = 3^22,23,26^) as there were not enough studies for gait or balance outcomes. For gait, results indicated no difference between nonstandard and standard PT (SMD, 0.03; 95% CI, −0.53 to 0.59). For balance and UPDRS, the results also indicated no significant difference between nonstandard and standard PT (balance: SMD, 0.54; 95% CI, −0.03 to 1.12; UPDRS: SMD, −0.49; 95% CI, −1.04 to 0.06). When comparing standard PT vs no intervention for UPDRS, results also lacked statistical significance (SMD, –1.09; 95% CI, −2.50 to 0.33). Across the 4 meta-analyses, between-study variance (τ^2^) ranged from 0.32 to 1.32, with most of this variance explained by actual differences rather than random error (*I*^2^ = 68%-88%). Forest plots are presented in Figure 2. Results of the meta-analytic moderator analyses were not statistically significant (eAppendix 4 in Supplement 1), and differences in outcomes by frequency of PT per week were not significant (SMD, 0.17; 95% CI, –0.03 to 0.36).

## Discussion

This comprehensive systematic review and meta-analysis revealed that many types of PT have shown benefit in the care of persons with PD. The study also revealed that there are gaps in our understanding of the effects of the type, timing, and frequency of PT for PD care, although maintenance of effect remains largely unexplored. Although a wide range of PT techniques and regimens have been commonly applied, there is scant head-to-head evidence comparing different techniques. A recently published expert viewpoint revealed no consensus on optimal timing and maintenance of PT for PD.^62^ Although many studies compared different interventions using comparative arms such as exercise therapy,^24,27,38,46,55^ few focused on uncovering the optimal implementation strategies for standard PT regimens with respect to type, timing, frequency, and maintenance of effect.^39^

Physical therapy has been established as an effective nonpharmacologic approach for the treatment of mobility deficits in patients with PD; however, there is a wide range of options. Complicating the choice is uncertainty on how exercise should be woven into implementation of PT. Previously published systematic reviews^11,12^ have focused on specific types of PT rather than the nature of the PT regimens. The 2 most common published outcomes in the current study were for gait (11 trials [24%]^23,25,26,28,30,31,32,42,44,53,61^) and balance (10 trials [22%]^18,25,29,33,43,44,48,52,53,58^). The evidence supported that different types of PT can be beneficial, and these ranged from general physiotherapy^16^ to specific tailored PT regimens.^42,43^ A wide range of PT durations, frequencies, and session lengths were applied across studies. Many PT regimens showed improvement in at least 1 area of physical function. Our findings aligned with the systematic review by Tomlinson et al (2012),^9^ which revealed benefits in speed and in BBS and UPDRS scores. The field of neurology has continued to be challenged with how to evaluate and control for home-based exercise in PT. For example, a systematic review and meta-analysis of exercise for PD published in 2021 showed that moderate aerobic exercise was associated with improved balance and gait; however, the outcomes for other PD motor symptoms were less clear.^10^ We deliberately focused our review on PT and not on exercise therapy. Our review included 5 trials with evidence supporting improvement in a primary outcome that was motor based.^17,27,40,49,52^ There were fewer studies showing benefits of other modalities such as dance^40^ and vestibular rehabilitation.^43^ This review addressed whether PT outcomes are durable after PT discontinuation, a largely unexplored topic in the literature. Many of the trials addressing this issue reported positive results on longer-term follow-up^18,23,24,29,31,32,36,37,40,41,42,44,47,49,52,56,58^; however, none of the trials followed up participants for more than 18 months.

Most trials in this study used PT regimens of 2 to 12 weeks with frequencies of 2 to 3 times weekly and with session lengths of 30 to 60 minutes. Although clinicians may consider this a reasonable standard when prescribing PT, without trial comparators, clinicians can only craft a reasonable guess. In addition, clinicians may be challenged to choose a therapy that will have the best chance for durability of outcome. Further complicating this is the nonsignificant results obtained from our meta-analytic moderator tests of duration, frequency, and number of sessions. Due to the limited number of studies amenable to meta-analysis, it remains unclear whether these factors were truly not associated with outcomes or whether findings were prone to type II error. More studies in this area may help to provide clarity.

The findings from this study may inform clinical practice and help physical therapists and physicians to implement PT for PD. Across geographical regions such as in the US, persons with PD can seek PT without a prescription from a physician. However, there are limited data for implementing gamepad-based training for balance^29^ or action observation treatment for freezing of gait.^31^ More comparative effectiveness research is needed. Also, many clinicians likely will not be aware of whether a facility offers specific interventions that require training or specialized equipment. Home-based exercises^46,48^ might improve access and adherence; however, few outcomes data are available on these regimens and how implementation could be weaved together with direct access to physical therapists or through physician prescriptions.

### Limitations

There were several limitations in this study. First, our search strategy might have missed publications addressing outcomes other than activities of daily living, quality of life, and/or motor outcomes. In addition, the diversity of tests used to measure outcomes rendered head-to-head comparisons challenging. One example of this limitation would be gait, which could be measured through speed,^28^ kinematic parameters,^23^ the timed up and go test,^44^ and the 10-m walk test.^29^ Although all these variables measure gait performance, each outcome should be interpreted as independent and specific to a clinical characteristic. Furthermore, pooling all trials and then reporting and analyzing all PT parameters for different PT types is a limitation. Ideally, one should aim for a better categorization of PT types and analysis of PT subcategories separately. However, this was not possible due to uncertain categorizations as well as numerous categories that each contained few studies. Another important limitation was the heterogeneity in PT types, timing, frequencies, and duration and the limited number of studies that compared these factors. As a result, our meta-analyses were based on a limited number of studies and thus were prone to type II error. More work needs to be done to confirm or refute our meta-analytic findings. Additionally, only a minority of trials included adequate long-term outcomes to assess durability of the intervention. Future studies should account for home exercise programs and their effects on outcomes.

## Conclusions

This systematic review and meta-analysis found that although the literature supports the use of PT as an effective treatment for PD and despite guidelines for inclusion of PT from the American Physical Therapy Association,^8^ implementation factors such as type, timing, frequency, and durability of outcomes remain largely unexplored. Our meta-analysis revealed no significant difference between standard PT and nonstandard PT for balance, gait, and UPDRS scores. These findings should be interpreted with caution and confirmed with better-powered studies. More controlled trials and comparative effectiveness studies are needed to evaluate the risks, benefits, and durability of each type of PT intervention and to guide better implementation.

## Notes

Supplement 1. **eAppendix 1.** Search Strategy Used to Retrieve Eligible Articles for the Review **eAppendix 2.** Summary of Systematic Review Findings **eAppendix 3.** Summary of the Risk of Bias in Trials Implementing an Intention-to-Treat Analysis and Trials Implementing a Per-Protocol Analysis **eAppendix 4.** Methods Used to Perform the Meta Moderators Analysis

Supplement 2.
Data Sharing Statement
