# Supplementary material for: Type, Timing, Frequency, and Durability of Outcome of Physical Therapy for Parkinson Disease: A Systematic Review and Meta-Analysis
Source: JAMA Netw Open. 2023 Jul 21;6(7):e2324860. doi: 10.1001/jamanetworkopen.2023.24860 (PMC10362470; doi:10.1001/jamanetworkopen.2023.24860)
Supplement: Supplement 1. — eAppendix 1. Search Strategy Used to Retrieve Eligible Articles for the Review eAppendix 2. Summary of Systematic Review Findings eAppendix 3. Summary of the Risk of Bias in Trials Implementing an Intention-to-Treat Analysis and Trials Implementing a Per-Protocol Analysis eAppendix 4. Methods Used to Perform the Meta Moderators Analysis [file jamanetwopen-e2324860-s001.pdf]

Supplementary Online Content

El Hayek M, Lobo Jofili Lopes JLM, LeLaurin JH, et al. Type, timing, frequency, and durability of outcome of physical therapy for Parkinson disease: a systematic review and meta-analysis. *JAMA Netw Open*. 2023;6(7):e2324860. doi:10.1001/jamanetworkopen.2023.24860

eAppendix 1. Search Strategy Used to Retrieve Eligible Articles for the Review
eAppendix 2. Summary of Systematic Review Findings
eAppendix 3. Summary of the Risk of Bias in Trials Implementing an Intention-to-Treat Analysis and Trials Implementing a Per-Protocol Analysis
eAppendix 4. Methods Used to Perform the Meta Moderators Analysis

This supplementary material has been provided by the authors to give readers additional information about their work.

© 2023 El Hayek M et al. *JAMA Network Open*.

eAppendix 1. Search Strategy Used to Retrieve Eligible Articles for the Review

PubMed via pubmed.ncbi.nlm.nih.gov/

#1 Search: "Parkinson Disease"[Majr]
#2 Search: "Parkinson Disease"[Majr] OR Parkinson's[text word]
#3. Search: "Parkinson Disease"[Majr] OR Parkinson's[tiab]
#4. Search: "Physical Therapy Modalities"[Majr]
#5. Search: "Physical Therapy Modalities"[Majr] OR physical therapy modalities[text word]
#6. Search: "Physical Therapy Modalities"[Majr] OR "physical therapy modalities"[text word]
#7. Search: "Time Factors"[Mesh]
#8 Search: timing[text word]
#9. Search: timing[tiab]
#10 Search: frequency[tiab]
#11 Search: "session spacing"[tiab]
#12 Search: "time factors"[tiab]
#13 Search: (("Activities of Daily Living"[Majr]) OR "Quality of Life"[Majr]) OR "Motor Skills"[Majr]
#14 Search: "quality of life"[tiab] OR "activities of daily living"[tiab] OR "motor skills"[tiab]
#15 Search: #14 OR #13
#16 Search: #7 OR #9 OR #10 OR #11 OR #12
#17 Search: #4 OR physical therapy modalit*[tiab]
#18 Search: #4 OR "physical therapy modalit*"[tiab]
#19 Search: #3 AND #15 AND #16 AND #18

Date Searched: August 10, 2022
Results: 48

Embase via Elsevier platform

('parkinson disease'/exp OR 'lewy bodies of parkinson disease' OR 'lewy bodies of parkinson`s disease' OR 'lewy bodies of parkinsons disease' OR 'lewy body parkinson disease' OR 'lewy body parkinson`s disease' OR 'lewy body parkinsons disease' OR 'parkinson dementia complex' OR 'parkinson disease' OR 'parkinson`s disease' OR 'parkinsons disease' OR 'idiopathic parkinsonism' OR 'paralysis agitans' OR 'primary parkinsonism')

AND

('physiotherapy'/exp OR 'physical therapy' OR 'physical therapy (speciality)' OR 'physical therapy (specialty)' OR 'physical therapy modalities' OR 'physical therapy service' OR 'physical therapy speciality' OR 'physical therapy specialty' OR 'physical therapy techniques' OR 'physical treatment' OR 'physio therapy' OR 'physiotherapy' OR 'physiotherapy department' OR 'therapy, physical')

AND ('time factor'/exp OR 'time factor' OR 'time factors' OR 'frequency'/exp OR 'frequency')

AND

('daily life activity'/exp OR 'adl (activities of daily living)' OR 'activities of daily living' OR 'activity, daily living' OR 'daily life activity' OR 'daily living activity' OR 'quality of life'/exp OR 'hrql' OR 'health related quality of life' OR 'life quality' OR 'quality of life' OR 'motor performance'/exp OR

© 2023 El Hayek M et al. *JAMA Network Open*.

'ability, motor' OR 'function, motor' OR 'motor ability' OR 'motor function' OR 'motor performance' OR 'motor skill' OR 'motor skills' OR 'performance, motor' OR 'skill, motor')

Date Searched: August 10, 2022
Results: 113

MEDLINE
Web of Science Core Collection
All of the above search via the Web of Science platform

AB=parkinson disease OR KP=parkinson disease

AND

AB=physiotherapy OR KP=physiotherapy OR AB="physical therapy" OR KP="physical therapy"

AND

AB=timing OR KP=timing OR AB=Frequency OR KP=frequency OR AB=delivery OR KP=delivery OR AB="spacing of sessions"

AND

AB="quality of life" OR KP="quality of life" OR AB="motor skills" OR KP="motor skills" OR AB="daily life activity" OR KP="daily life activity" OR AB=rehabilitation OR KP=rehabilitation OR AB="activities of daily living" OR KP="Activities of daily living" OR AB=emotions OR KP=emotions OR AB=anxiety OR KP=anxiety OR AB=depression OR AB=happiness

Date Searched: August 10, 2022
Results: 212

© 2023 El Hayek M et al. *JAMA Network Open*.

eAppendix 2.

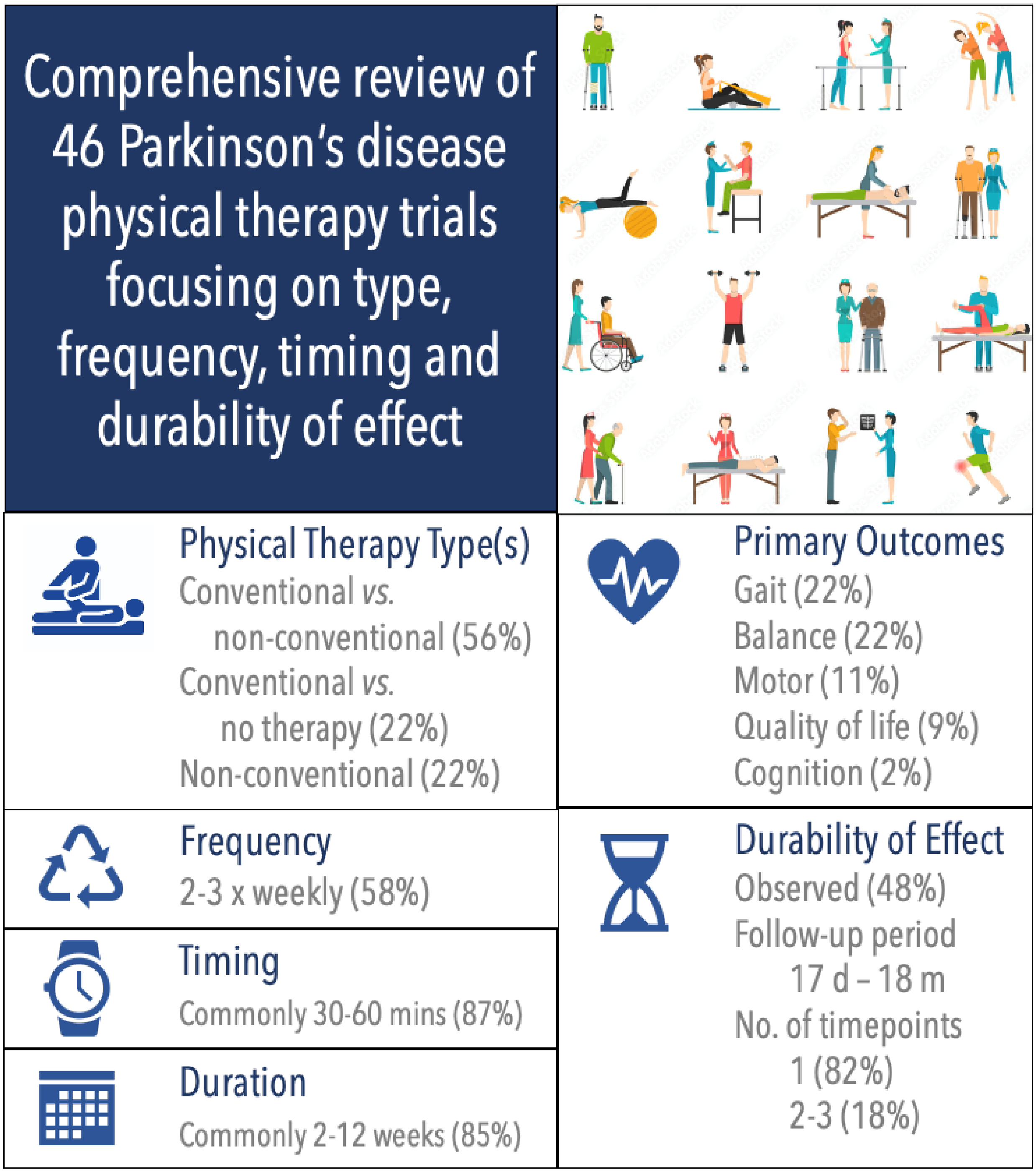

eAppendix 2. Summary of systematic review findings

© 2023 El Hayek M et al. *JAMA Network Open*.

eAppendix 3

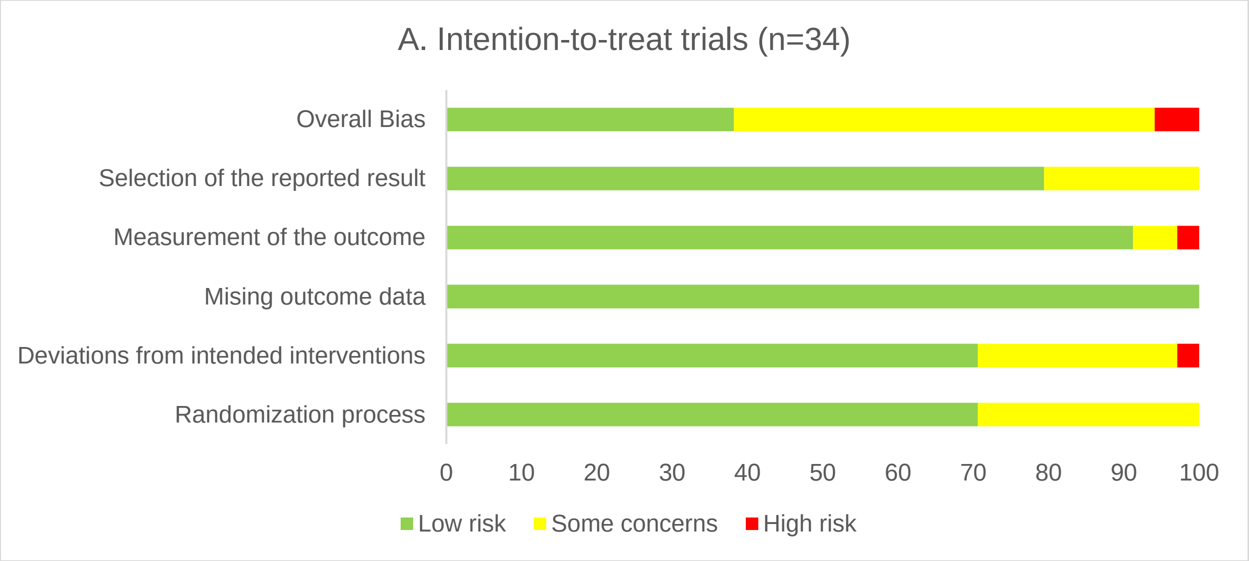

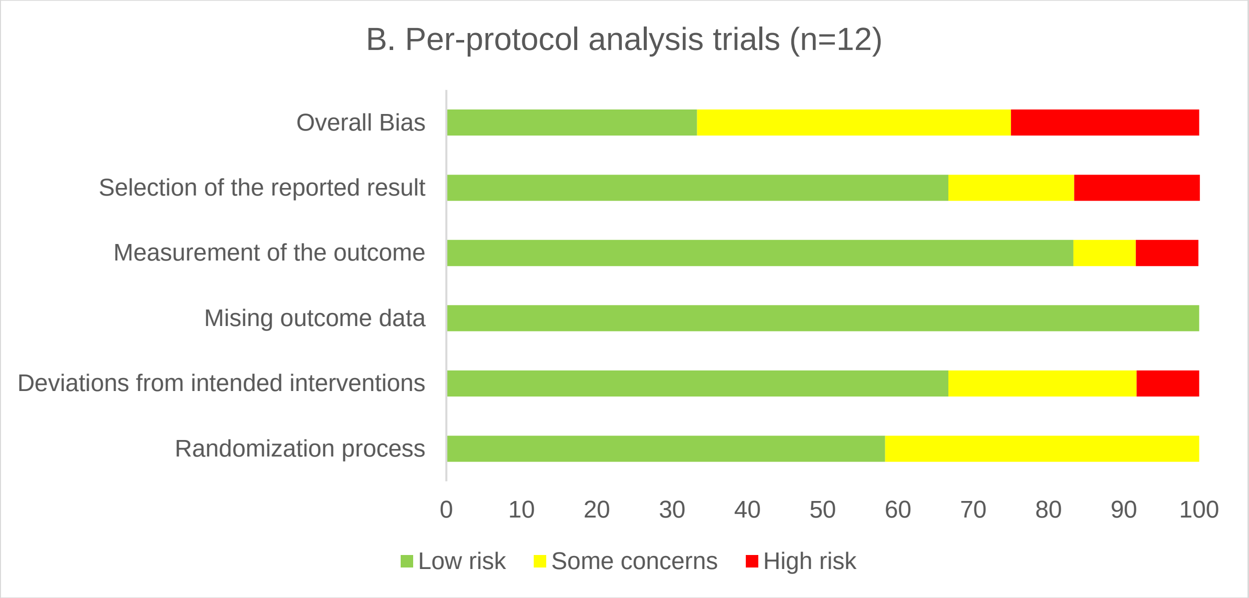

eAppendix 3. Summary of the risk of bias in (A) trials implementing an intention-to-treat analysis (n=34) and (B) trials implementing a per-protocol analysis (n=12).

© 2023 El Hayek M et al. *JAMA Network Open*.

eAppendix 4. Methods Used to Perform the Meta Moderators Analysis

Because we were insufficiently powered to conduct meta-analytic moderator tests within each of the four meta-analyses we conducted, we combined all of the studies in the meta-analyses to test moderators. Across all 4 meta-analyses conducted, there were 26 effect sizes from 16 studies. Using a mixed-effects model with Restricted Maximum Likelihood estimation in the metafor package in R[61], we set study ID as a random factor so that each study was only included in the meta-analytic moderator models once.

In the meta-analytic dataset, the following ranges were present: duration = 4-24 weeks; frequency per week = 0.5-7; total number of sessions = 6-60. For continuous analyses, we centered these variables so that zero was a plausible value. We ran each moderator independently. Duration was not significant (SMD = 0.00, 95% CI = -0.06, 0.06), nor was frequency per week (SMD = 0.17, 95% CI = -0.03, 0.36) or total number of sessions (SMD = 0.02, 95% CI = -0.01, 0.04). Dichotomized duration (<12 and ≥12 weeks) was similarly not significant (SMD = -0.12, 95% CI = -0.95, 0.72), nor was the model comparing ≤6 and >6 weeks (SMD = -0.03, 95% CI = -0.75, 0.81).

© 2023 El Hayek M et al. *JAMA Network Open*.
