# Supplementary material for: Type, Timing, Frequency, and Durability of Outcome of Physical Therapy for Parkinson Disease: A Systematic Review and Meta-Analysis
Source: JAMA Netw Open. 2023 Jul 21;6(7):e2324860. doi: 10.1001/jamanetworkopen.2023.24860 (PMC10362470; doi:10.1001/jamanetworkopen.2023.24860)
Supplement: Supplement 2. — Data Sharing Statement [file jamanetwopen-e2324860-s002.pdf]

Data Sharing Statement

El Hayek. Type, Timing, Frequency, and Durability of Outcome of Physical Therapy for Parkinson Disease. *JAMA Netw Open*. Published July 21, 2023.
doi:10.1001/jamanetworkopen.2023.24860

Data
Data available: No
